# Supplementary material for: Bayesian refinement of protein structures and ensembles against SAXS data using molecular dynamics
Source: PLoS Comput Biol. 2017 Oct 18;13(10):e1005800. doi: 10.1371/journal.pcbi.1005800 (PMC5662244; doi:10.1371/journal.pcbi.1005800)
Supplement: S1 Archive — (BZ2) [file pcbi.1005800.s011.tar.bz2 › gromacs_4.62_bayesian_saxs_refi/share/html/online/tpb.html]

tpb file format


## tpb file format

|  |  |
| --- | --- |
| Main Table of Contents | **VERSION 4.6** |
| GROMACS homepage | **Sat 19 Jan 2013** |

---

### Description

The tpb file extension stands for binary run input file. This file contains
the starting structure of your simulation, The molecular topology and
all the simulation data. Because this file is in binary format it
cannot be read with a normal editor. To read a binary run input file type:

```
% gmxdump -s topol.tpb
```

or if you're not such a fast reader:

```
% gmxdump -s topol.tpb | more
```

You can also compare two tpb files using:

```
% gmxcheck -s1 top1 -s2 top2 | more
```

|  |  |
| --- | --- |
| Main Table of Contents | **VERSION 4.6** |
| GROMACS homepage | **Sat 19 Jan 2013** |

---
